# Prevention of Ocular Scarring Post Glaucoma Filtration Surgery Using the Inflammatory Cell and Platelet Binding Modulator Saratin in a Rabbit Model

**DOI:** 10.1371/journal.pone.0035627

**Published:** 2012-04-30

**Authors:** Jeff Min, Zachary L. Lukowski, Monica A. Levine, Craig A. Meyers, Ashley R. Beattie, Gregory S. Schultz, Don A. Samuelson, Mark B. Sherwood

**Affiliations:** 1 Department of Ophthalmology, College of Medicine, University of Florida, Gainesville, Florida, United States of America; 2 Department of Ob/Gyn and Institute of Wound Healing, College of Medicine, University of Florida, Gainesville, Florida, United States of America; 3 College of Veterinary Medicine, University of Florida, Gainesville, Florida, United States of America; Massachusetts Eye & Ear Infirmary, Harvard Medical School, United States of America

## Abstract

**Clinical Relevance:**

Late complications can occur with use of current antimetabolites to prevent scarring following glaucoma filtration surgery (GFS). Safer, more targeted, anti-fibrosis agents are sought.

**Objectives:**

The protein saratin has been shown to exhibit anti-fibrotic and anti-thrombotic properties in response to injury, but had not been used for glaucoma surgery. The goal of this study was to compare the efficacy of saratin with that of the widely accepted mitomycin-C (MMC) in prolonging bleb survival following GFS in the rabbit model. Two saratin delivery routes were compared; a single intraoperative topical application versus a combination of intraoperative topical application with two additional postoperative injections.

**Methods:**

Twenty-four New Zealand White rabbits underwent GFS and received either intraoperative topical saratin, intraoperative topical saratin plus two injections on post-operative days 4 and 8, balanced saline solution (BSS), or MMC. The bleb tissues and their elevation durations were compared based on clinical and histological findings.

**Results:**

Rabbits receiving topical+injections of saratin had a mean bleb survival of 33.6±8.5 days, significantly higher than the negative BSS controls, which averaged 17.4±6.0 days (p = 0.018). No improvement over BSS was seen for rabbits receiving topical saratin only (15.5±4.8 days, p = 0.749). Rabbits receiving saratin did not develop bleb avascularity and thinning associated with MMC treatment and there were no apparent clinical signs of toxicity.

**Conclusions:**

Treatment with a single intraoperative topical application plus two additional postoperative injections significantly prolonged bleb elevation comparable to MMC, but without toxicity; however, topical application alone was ineffective.

## Introduction

Glaucoma filtration surgery (GFS) remains the gold standard for the management of intraocular pressure (IOP) when medication and laser surgery have proven insufficient [1], [2]. However, scar tissue due increased fibroblast proliferation and activation can form between the conjunctiva/Tenon’s capsule and the sclera at the surgical site often months or years after surgery, obstructing aqueous flow and causing the filter to fail [3], [4].

The antimetabolites mitomycin-C (MMC) and 5-fluorouracil (5-FU) are used in current clinical practice to help limit post-operative ocular scar tissue formation. While these agents have been shown to improve the IOP outcome of GFS, they do so in a non-selective manner [5]. As a result, antimetabolite treatment is associated with a significant side-effect profile, including hypotony, blebitis, endophthalmitis, bleb leakage, and vision loss in a few [2], [3], [5]–[8]. Recent studies into alternative methods of preventing tissue fibrosis have focused on the inhibition of Tenon’s capsule fibroblasts through the regulation of various growth factors [9]–[11]. Some success in reducing scar formation after GFS by inhibiting vascular endothelial growth factor (VEGF) and transforming growth factor-β (TGF-β) has been reported in recent literature [5], [12], [13].

The focus of this investigation is to examine the ability of saratin to limit ocular scarring following GFS. Saratin is a 12 kD protein which was originally isolated from the saliva of the leech *Hirudo medicinalis*. It has been shown to interfere with platelet integrin α_2_β_1_-collagen and von Willebrand factor-collagen binding, preventing platelet aggregation in response to injury [14]–[17]. To date, saratin has been shown to be effective in preventing platelet adhesion and intimal hyperplasia in both animal and human models [18], [19]. Recent studies in human models have shown that the protein has potent antithrombotic properties, making it useful in the treatment of atherosclerotic lesions [20].

Evidence also suggests that saratin can potentially reduce inflammation by limiting the immune response at the site of tissue injury. Primarily, it is thought that the protein interferes with the binding of inflammatory cells to extracellular matrix proteins, a necessary step for cell migration toward wound sites [21]. *In vitro* Boyden chamber assays indicated that saratin inhibits the chemotaxis of human monocytes and T-lymphocytes toward the chemokines MCP-1 and MIP-1α, respectively [22]. Secondarily, by interfering with platelet aggregation at the wound site, saratin limits the release of platelet-derived growth factor (PDGF), TGF-β, insulin-like growth factor (IGF), and epidermal growth factor (EGF) [23]. These growth factors have been shown to contribute toward accelerated ocular wound healing by stimulating proliferation, migration, and collagen production in Tenon’s capsule fibroblasts [10], [11], [24], [25].

The rabbit model is well-established as a standard animal model for the investigation of wound healing and scarring following GFS [25]–[28]. Our experiment investigates the ability of saratin to reduce fibrotic activity at the site of GFS. To our knowledge, this is the first reported ocular use of the saratin protein. The primary goals of this study were to evaluate potential methods of delivering saratin, as well as to compare the overall efficacy of saratin versus a BSS negative control and a MMC positive control. An additional aim for this project was to identify potential side effects of treatment using saratin.

## Methods and Materials

Twenty four New Zealand White rabbits weighting between 2 kg and 4 kg were used for this study. All animal experiments performed adhered to the ARVO Statement for the Use of Animals in Ophthalmic and Vision Research and were approved by the University of Florida’s Institutional Animal Care and Use Committee.

### Study Design

The rabbits were randomized to one of 4 different treatment groups of six rabbits each. All 24 rabbits underwent glaucoma filtering operation in their left eyes with one surgeon (MBS) performing the procedures; the right eyes were not operated on and served as controls.

Rabbits in the first experimental group received a single topical application of saratin at 5 mg/ml applied intraoperatively using a standardized 5 mm×5 mm partial-thickness Weck cell (Alcon Surgical, Fort Worth, TX), immediately following the fashioning of a fornix-based conjunctival flap.

Rabbits in the second experimental group also received a topical application with the partial-thickness Weck cell sponge at the time of surgery. In addition, two 0.1 ml subconjunctival injections of saratin at 5 mg/ml were administered on post-operative days (POD) 4 and 8. The protein saratin used in both experimental groups one and two was supplied by BioVascular, Inc., is now made by recombinant expression in yeast (*Hansenula polymorpha*) and purified to greater than 95% purity.

To provide a more accurate comparison to the experimental groups, all rabbits in the negative control group received an intraoperative topical application of BSS. Three (half) of the rabbits in this group also received 0.1 ml subconjunctival injections of BSS on POD 4 and 8 to match group 2 rabbits and three did not in order to mimic group 1 rabbits and MMC positive controls. All postoperative subconjunctival injections were given following bleb examination by the masked observer, and the injection material remained unknown to both the observer and the clinician giving the injection.

Finally, rabbits in the positive control group received a topical application of 0.4 mg/ml mitomycin-C at the time of surgery and were not given any post-operative injections.

### Glaucoma Filtering Operation

The rabbits were anesthetized with an intramuscular injection of a combination of 50 mg/kg ketamine (“Ketaject”, Phoenix, Mo) and 10 mg/kg xylazine (“Xyla-ject”, Phoenix, Mo). A topical anesthetic, 0.1% proparacaine eye drop (Bausch & Lomb, Tampa, FL), was also administered. The surgical technique for the glaucoma filtration operations was similar to the procedures described in previous publications [25]. In brief, an eyelid speculum retracted the eyelids. A partial-thickness, corneal traction suture made in the superior cornea rotated the eye inferiorly. In the superior lateral quadrant of the eye at the limbus, a measured standard sized 5 mm fornix-based conjunctival flap was created. Blunt dissection helped undermine the conjunctiva and Tenon’s capsule. At this point, a 5 mm×5 mm partial-thickness Weck cell (Alcon Surgical, Fort Worth, TX) soaked in 5 mg/ml saratin, 0.4 mg/ml mitomycin-C, or BSS depending on the treatment group was placed locally between the conjunctiva/Tenon’s capsule and the sclera. After 5 minutes, the sponge was removed and the area was washed with 30 ml of saline. Next, a clear corneal paracentesis tract was fashioned in the superonasal quadrant using a # 75 Beaver™ blade (Becton Dickinson & Co., Franklin Lakes, NJ). A viscoelastic material (Healon® 10mg/ml, Pharmacia & Upjohn) was injected to maintain the anterior chamber.

A 25-gauge needle was then used to create a beveled, full-thickness tract through the sclera into the anterior chamber about 1 mm posterior to the limbus. This was followed by insertion of a 22-gauge, IV cannula (Insyte® Becton Dickinson Vascular Access, Sandy, UT) along this tract into the anterior chamber. The cannula needle was pulled out, and the cannula was positioned beyond the pupillary margin to prevent the tube from being occluded by iris. The cannula was trimmed at its scleral end so it would not protrude more than approximately 1 mm from the point of insertion. A 10–0 nylon suture (Ethicon Inc., Somerville, NJ) was used to anchor the tube to the sclera.

A running suture of 8–0 absorbable suture material (*Vicryl*®, Ethicon Inc., Somerville, NJ) was used to close the conjunctiva/Tenon’s capsule at the limbus in a watertight fashion. At the end of the procedure, saline was injected into the anterior chamber via the paracentesis tract to elevate the bleb and a Seidel test performed to ensure that there was no bleb leak. An ointment of neomycin and dexamethasone was applied topically immediately following the surgery.

### Post-Operative Injections and Clinical Evaluations

Following the operation, the eyes were examined three times per week by an examiner masked to the treatment group. The examiner looked for the presence of bleb elevation as well as for any complications that may have resulted from the surgeries or various treatments, including conjunctival injection, edema, anterior chamber shallowing, tube mal-positioning, hyphema or sub-conjunctival hemorrhage, bleb leaks, and corneal or lens opacification. Bleb failure was declared after the masked observer deemed the bleb to be flat in two consecutive examinations. The first of the two dates was recorded as the endpoint. When applicable, rabbits in the saratin and BSS treatment groups were also given injections of 0.1 ml saratin or 0.1 ml BSS respectively on POD 4 and 8.

The rabbits were anesthetized with isoflurane during their post-operative examinations, and a topical 0.1% proparacaine anesthetic eye drop was administered for those that required a post-op injection. A speculum was used to retract the eyelids. Non-toothed Bishop-Harmon forceps were used to tent the conjunctiva, and the 0.1 ml injections were given near the bleb using a 30-gauge needle on a 1 ml syringe on POD 4 and 8.

### Histology

Eyes were obtained from one rabbit in each of the four groups on post-operative day (POD) 12 so bleb tissues could be examined at a uniform time. Eyes were obtained from all other rabbits after the bleb was observed to be flat in two consecutive clinical evaluations.

The tissues were fixed for 24 hours in a 10% neutral buffered formalin solution. The globes were placed in tissue cassettes, and then processed through graded ethanol and xylene using a Sakura Tissue-Tek VIP 5 tissue processor (Torrance, CA). The tissues were infiltrated with paraffin (Richard-Allan Scientific, Kalamazoo, MI) and embedded on a Tissue Tek III embedding center. Sagittal serial sections of the globes were taken. Once deparaffinized, the sections were stained using either standard Harris hematoxylin and eosin or Masson’s trichrome. The slides were examined under light microscopy by an observer masked to the treatment groups and representative sections were photographed using a Canon EOS T1i digital camera attached to an Olympus Vanox microscope. The slides were qualitatively graded based upon collagen and fibroblast densities and vascularity in the tissues around the end of the cannula, plus preservation of the conjunctival tissue morphology including goblet cell numbers.

### Statistical Analysis

Analysis of Variance (ANOVA) testing was used to determine any significant difference in bleb survival duration among the treatment groups. Tukey’s Honestly Significantly Different (HSD) post hoc test was then used to determine significance between pairs of relevant groups.

## Results

### Bleb Survival

**Figure 1:**
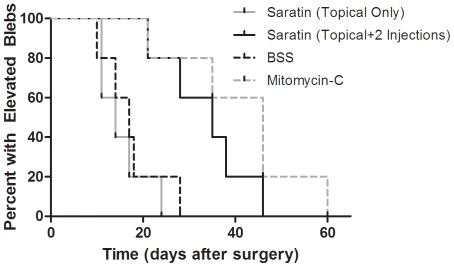
Kaplan-Meier bleb survival plot of eyes in each treatment group. For each rabbit, bleb failure was declared after the bleb appeared flat in two consecutive masked clinical examinations. The first of the two dates was recorded as the endpoint.

**Figure 2:**
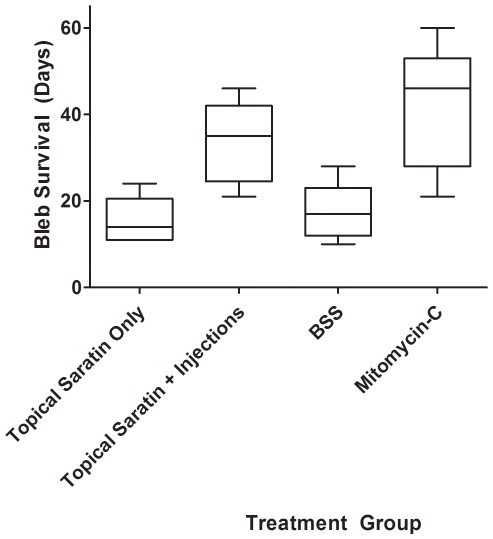
Box-and-whisker plot of bleb survival for each treatment group. First and third quartiles are delineated by boxes. Whiskers represent maxima and minima.

Rabbits receiving a single 5 minute topical application of saratin had an average time to bleb failure of 15.5^+^/– 4.8 days. Rabbits receiving two additional injections of saratin on POD 4 and 8 had an average time of 33.6^+^/– 8.5 days. The average time to bleb failure for rabbits receiving BSS injections/topical application was 17.4^+^/– 6.0 days. Rabbits receiving MMC treatments had bleb elevation durations averaging 41.6+/– 13.0 days. The rabbits from each treatment group sacrificed on POD 12 for histologic comparison were omitted from these data calculations. These results are represented in a Kaplan-Meier survival plot (Fig. 1) and summarized using a box-and-whisker plot (Fig. 2).

Analysis of variance (ANOVA) testing revealed a significant difference among the treatment groups (p = 0.001) at a 95% confidence interval. Post-hoc testing with Tukey’s Honestly Significant Different (HSD) showed that the MMC rabbit group had a significantly longer bleb survival duration than the BSS group at a 95% confidence interval. In addition, there was a significant difference in bleb survival duration between the topical+injection saratin group and the BSS group at a 90% confidence interval. No significant improvements over BSS were found for the topical-only saratin group at a 90 or 95% confidence interval. Finally, there was no statistically significant difference between the MMC group and the topical+injection saratin treatment group at a 90% or 95% confidence interval.

### Clinical Evaluation

Blebs treated with MMC developed avascularity that was not observed in either saratin or BSS groups. There were no clinical signs of obvious toxicity to saratin treatments.

### Histology

**Figure 3:**
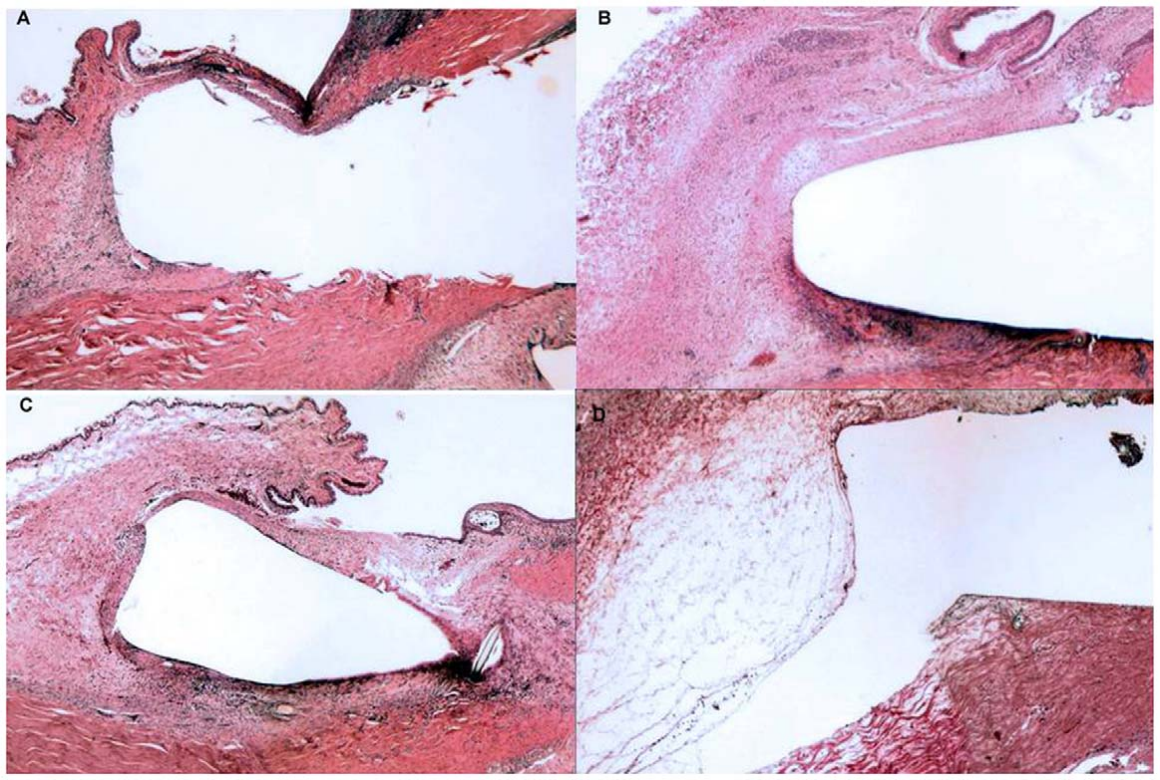
Representative sections of the implant site taken on POD 12, H&E 20x; (A) Topical saratin; (B) Topical+2 injections of saratin; (C) Balanced saline solution; (D) Mitomycin-C.

**Figure 4:**
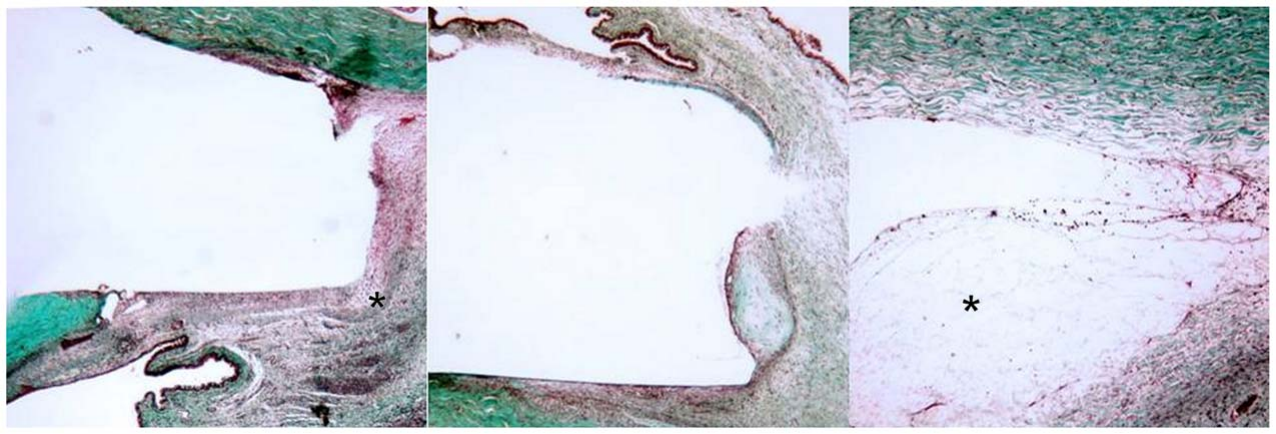
Representative sections of the implant site taken on POD 12, Masson’s Trichrome 20x; Left: Topical+2 injections of saratin; *asterisk*, mild collagen density and moderate fibroblast proliferation Middle: Balanced saline solution; Right: Mitomycin-C; *asterisk*, acellular fibrosis and necrosis.

**Figure 5:**
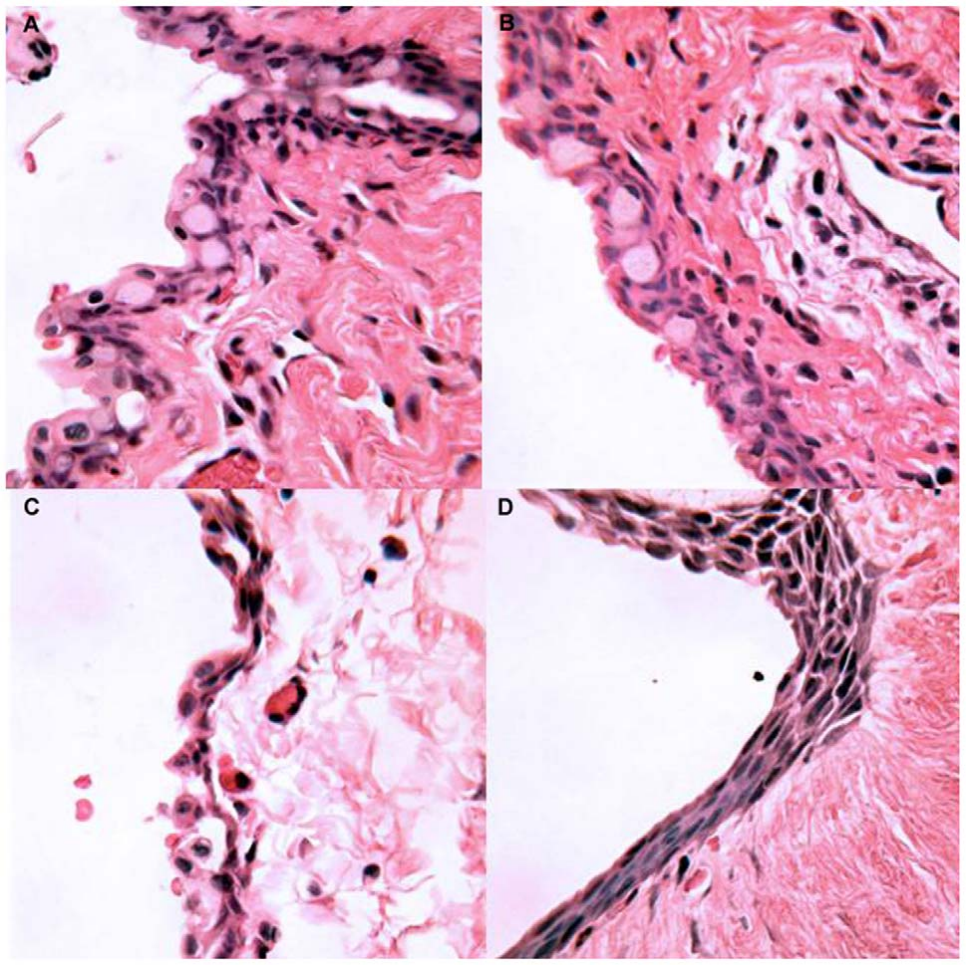
Representative sections of the bleb conjunctiva taken on POD 12, H&E 250x; (A) Topical saratin; (B) Topical+2 injections of saratin; (C) Balanced saline solution; (D) Mitomycin-C.

Histological examination focused on samples taken on post-operative day 12 to provide a common time-point of comparison (Fig. 3 & 4). Among all treatment groups, a zone of fibroblast proliferation with associated intracellular collagen was observed bordering the site of the implant. A variable infiltration of plasma cells and polymorphonuclear leukocytes (PMNLs) was evident within this area. The rabbits that received saratin by either topical application or injections had a mild collagen density and a moderate fibroblast proliferation. The group that received BSS showed a mild to moderate fibroblast density and a collagen density similar to the animals in the saratin group. The MMC group showed slightly less fibrosis, with minimal to mild collagen and fibroblast density highlighted by a large region of acellular fibrosis and necrosis (Fig. 4, right). The conjunctiva of rabbits treated with saratin did not exhibit avascularity, tissue thinning, and change in epithelial morphology from pseudostratified columnar to stratified squamous with a loss of mucin-producing (goblet) cells typical of MMC treatment (Fig. 5).

## Discussion

In this study we examined the antifibrotic effects of saratin, a protein which has been previously investigated as an antithrombotic agent. Because the application of saratin in GFS was a novel use for the protein, we tested two methods of delivering the drug. The first experimental group received only an intraoperative topical application similar to the method in which MMC is currently administered. A second experimental group received a combined treatment of topical saratin with additional subconjunctival injections on POD 4 and 8. Because the amount of saratin retained in ocular tissues is unknown, the injections in the latter treatment modality ensured high amounts of the protein over an extended duration.

Statistical analysis showed that intraoperative topical saratin alone did not improve bleb survival compared to BSS negative controls. The Kaplan-Meier survival plot (Fig. 1) and box-and-whisker plot (Fig. 2) show that rabbits in the topical saratin group performed similarly to those in the BSS group. However, the group which received postoperative injections of saratin in addition to topical application had a significant improvement over BSS negative controls and was comparable to the MMC positive control group. While the mean for the topical+injection saratin group was slightly less than that of the MMC group (33.6^+^/– 8.5 days vs. 41.6+/– 13.0 days), the difference was not statistically significant.

When applied topically for a brief period of 1–5 minutes, MMC has an immediate effect on local tissues by alkylating and crosslinking DNA, as well as inhibiting DNA synthesis, transcription, and translation [29]. Topical saratin, when administered in a similar manner for 5 minutes did not improve bleb survival beyond that of the BSS control. When two post-operative subconjunctival injections were given in addition, there was a significant improvement over BSS control and the bleb survival was not significantly different from the MMC positive control. The ineffectiveness of topical saratin when administered at surgery using a partial-thickness Weck cell sponge suggests three possibilities; either a lack of local uptake of the saratin from the sponge, or that contact with the tissues was not long enough to produce an effect, or that peroperative application is not the most effective time to deliver saratin compared to early post-operative treatment. The optimal delivery route, number of applications, and the half-life of saratin in the ocular tissues warrant further study.

Clinically and histologically, saratin did not appear to cause bleb avascularity and tissue thinning, including alteration in epithelial structure, commonly associated with MMC treatment. While the topical+injection group did not outperform MMC, the effects of MMC have been extensively studied and the dosage optimized [2], [3], [5]–[8], [29]. Further studies on saratin may be necessary to improve the dosing regimen and to provide additional information regarding potential side-effects.
